# Molecular interplay between ecdysone receptor and retinoid X receptor in regulating the molting of the Chinese mitten crab, *Eriocheir sinensis*

**DOI:** 10.3389/fendo.2023.1251723

**Published:** 2023-10-19

**Authors:** Xiaowen Chen, Xin Hou, Hao Yang, Hao Liu, Jun Wang, Chenghui Wang

**Affiliations:** Key Laboratory of Freshwater Fisheries Germplasm Resources Certificated by The Ministry of Agriculture and Rural Affairs/National Demonstration Center for Experimental Fisheries Science Education/Shanghai Engineering/Research Center of Aquaculture, Shanghai Ocean University, Shanghai, China

**Keywords:** molting, ecdysteroid signaling pathway, molecular interplay, ecdysone receptor, retinoid X receptor

## Abstract

**Purpose:**

Molting is a pivotal biological process regulated by the ecdysteroid signaling pathway that requires molecular coordination of two transcription factors, Ecdysone receptor (EcR) and ultraspiracle (USP) in arthropods. However, the molecular interplay of EcR and Retinoid X receptor (RXR), the crustacean homolog of USP in the ecdysteroid signaling pathway, is not well understood.

**Methods:**

In this study, we conducted temporal and spatial expression, co-immunoprecipitation (CO-IP), and luciferase reporter assay experiments to investigate the molecular function and interplay of EcR and RXR during the molting process of the Chinese mitten crab, *Eriocheir sinensis*.

**Results:**

The results showed that the expression level of *RXR* was more stable and significantly higher than *EcR* during the entire molting process. However, the expression level of *EcR* fluctuated dynamically and increased sharply at the premolt stage. The CO-IP and luciferase reporter assay results confirmed the molecular interplay of EcR and RXR. The heterodimer complex formed by the two transcription factors significantly induced the transcription of *E75*, an essential gene in the ecdysteroid signaling pathway.

**Conclusions:**

Our study unveiled the diverse molecular function and molecular interplay of EcR and RXR; *RXR* is possibly a “constitutive-type” gene, and *EcR* is possibly a vital speed-limiting gene while both *EcR* and *RXR* are required to initiate the ecdysteroid signaling cascade, which may be indispensable for molting regulation in *E. sinensis.* The results provide a theoretical basis for the endocrine control of molting in *E. sinensis* and novel insights into the molecular mechanism of molting mediated by the ecdysteroid signaling pathway in crustaceans.

## 1 Introduction

Molting plays essential roles in growth and reproduction, which is regulated by the ecdysteroid signaling pathway in arthropods (1). The ecdysteroid signaling pathway is a signal cascade process that requires molecular regulation of two transcription factors, ecdysone receptor (*EcR*) and ultraspiracle (*USP*), ecdysteroid, and other factors (2). During the molting process, the response to ecdysteroid is mediated by a heterodimer complex receptor composed of USP and EcR (3), which can initiate the transcriptional activation of a cascade of downstream genes and induce the subsequent molting (4–7). In crustaceans, *RXR* is the homologous gene of *USP* in insects, and it is believed to have similar molecular functions in regulating molting (2). In insects, it has been reported that EcR binds to USP to form heterodimer complex, and then ecdysteroid binds to the heterodimer complex to activate the transcription of downstream genes in the ecdysteroid cassette signaling pathway (6–11). However, the extent to which RXR interacts with EcR in regulating molting in crustaceans remains incompletely understood.

While studies have revealed that the crystal structure of the heterodimer formed by EcR and RXR in crustaceans may resemble that of the EcR/USP complex in insects, some differences in structure and function have been identified between RXR and USP (12). The *in vitro* experiments of CV1 cells indicated the EcR/RXR heterodimer has different pharmacological and functional properties from the EcR/USP heterodimer (5). In CV1 and Hela cells, it was observed that the DNA binding activity of EcR/USP remained unaffected by hormones, whereas the DNA binding activity of EcR/RXR was found to be influenced by ecdysteroids or 9-cis retinoic acid (13). Juvenile hormones can directly regulate the activity of the EcR/USP heterodimer complex in insects, but exogenous 9-cis-retinoic acid and juvenile hormones are not able to activate EcR/RXR complex in cultured Sf 9 cells (14). Compared with RXR, USP is more effective and can lead to the nuclear localization EcR even without the ligands (15). Furthermore, it has been observed that USP is insensitive to the known RXR ligands, which suggests that the function of USP may differ from that of RXR (16, 17). As yet, it remains unclear whether EcR forms a heterodimer complex with RXR in crustaceans, and whether the interaction between EcR and RXR in crustaceans is analogous to that observed in insects.

Moreover, it has been indicated that EcR and USP can bind with the ecdysteroid response elements of the ecdysteroid inducible gene *E75*, *Hsp27*, *Dronc*, and other genes, thereby initiating their transcription (18–21). *E75*, *E74*, and *BrC* genes are the early responsive genes of the ecdysteroid signaling cascade pathway and play crucial roles in the molting process (22). However, in crustaceans, it has yet to be definitively established whether the classical ecdysteroid signaling cascade pathway, as inferred from insect research that EcR forms a heterodimer complex with RXR to initiate the transcription of early responsive genes such as *E75* during molting, is applicable.

*Eriocheir sinensis*, belonging to the Arthropoda, Crustacca, Decapoda, Grapsidae, and Eriocheir genera, is widely distributed in lakes along the north and south coasts of China and one of the most important aquaculture and economic crabs in China (23). In its whole life, periodic molts will occurs 18-21 times and the ecdysone signaling pathway regulate the molting process (24). In this study, we deciphered the gene structure of *EcR* and *RXR*, conducted spatial and temporal expression experiments, co-immunoprecipitation, and luciferase reporter assay experiments of these two genes in *E. sinensis*. Our objective was to investigate the molecular function and molecular interplay of EcR and RXR in the molting regulation of *E. sinensis.*

## 2 Materials and methods

### 2.1 Sample collection and ethics statement

In this study, all the *E. sinensis* individuals were collected from the Aquatic Animal Germplasm Station of Shanghai Ocean University (Shanghai, China). The molting stage was observed and determined according to the setal developmental characters of the second maxilla (25). The hepatopancreas, muscle, and epidermis tissues were sampled at the premolt (PrM), intermolt (InM), and postmolt (PoM) stages with six biological replicates. Sampling procedures complied with the guidelines of the Institutional Animal Care and Use Committee (IACUC) of Shanghai Ocean University on the care and use of animals for scientific purposes. The experimental protocols were approved by the IACUC of Shanghai Ocean University.

### 2.2 RNA isolation and cDNA synthesis

Total RNA from each tissue was extracted using a MiniBEST Universal RNA Extraction Kit (Takara, Dalian, China) according to the manufacturer’s instructions. The quality of the extracted RNA was evaluated by 1.0% agarose gels and the quantity of RNA was measured by NanoDrop 2000 (Thermo Fisher, USA). A total of 1 μg RNA was used to synthesize complementary DNA (cDNA) using the PrimeSript™ RT reagent Kit (Takara, Dalian, China) in accordance with the manufacturer’s protocol.

### 2.3 Gene structure, cloning and sequence alignment

The gene sequence of *RXR* and exon-intron information was extracted from the assembled genome of *E. sinensis* (26). The gene structure of *RXR* was visualized using the gene structure display server 2 (http://gsds.gao-lab.org/) (27). To verify the open reading frame (ORF) sequence of *RXR*, primer was designed according to the extracted mRNA sequence of *RXR* (**Table S1**), and PCR was performed on the BioRad Thermal Cycler (California, USA). The expected PCR products were sequenced with an ABI3730 sequencer (Sangon, Shanghai, China). The amino acid sequence of RXR was translated according to the verified ORF sequence, and the protein domains were predicted using the CD-search software with the NCBI Conserved Domain Database (https://www.ncbi.nlm.nih.gov/Structure/cdd/cdd.shtml) (28). The amino acid sequences of RXR from *Callinectes sapidus* (HQ630860.1), *Fenneropenaeus chinensis* (FJ194479.1), *Daphnia magna* (DQ530508.1)*, Drosophila melanogaster* (NM_057433.4), *Homo sapiens* (U38480.1), and *Mus musculus* (M84817.1) were downloaded from NCBI database. Multiple sequence alignment was conducted using Clustal X software.

### 2.4 Construction of phylogenetic tree and prediction of protein structure

A phylogenetic tree was constructed by MEGA 11 software using maximum likelihood methods with 1,000 bootstraps based on the amino acid sequence of *RXR* open reading frame and the amino acid sequence of *EcR* was used as outgroup. The nucleotide sequences of *RXR* from *M. musculus* (NM_011305.3), *H. sapiens* (NM_001291920.2), *Danio rerio* (NM_131217.4), *Salmo salar* (XM_014132462.2), *Oncorhynchus mykiss* (XM_036977326.1), *D. magna* (AB274819.1), *Portunus trituberculatus* (KF061043.2), *Scylla paramamosain* (KT970086.1), *Penaeus monodon* (KX898397.1), *Litopenaeus vannamei* (KF234773.1), *Fenneropenaeus chinensis* (FJ194479.1), *Manduca sexta* (U44837.1), *Bombyx mori* (NM_001044005.1), *Spodoptera litura* (JQ730734.1), *Bactrocera dorsalis* (HM195185.1) involved in the construction of the phylogenetic tree were downloaded from NCBI database. The 3D protein structures of EcR and RXR were modeled by the Swiss-Model server (https://swissmodel.expasy.org/) based on 4nqa.1. B and 4nqa.1.A as template, respectively (29).

### 2.5 Relative expression profiles of *EcR* and *RXR* during the molting cycle

Quantitative real-time PCR (qRT-PCR) was used to quantify the relative expression levels of *EcR* and *RXR* in hepatopancreas, muscle, and epidermis during different molting stages. Beta-actin (*β-actin*) was used as the normalized gene for qRT-PCR (30). The primer for *RXR* was designed according to the verified gene sequence and the primer for *EcR* was designed according to our previously published paper (**Table S1**) (2). The amplification efficiency and specificity of primers were checked for validation, and the primers used in this study had an amplification efficiency between 95% and 105%. qRT-PCR for *RXR* and *EcR* was performed with a 25 μL reaction mixture that contained 12.5 μL SYBR Green Premix Ex Taq (Takara, Dalian, China), 1 μL primer (10 μM), 2 μL dilution cDNA, and 8.5 μL dd H_2_O. The PCR procedure was as follows: 95°C for 30 s, followed by 40 cycles of 95°C for 5 s, and 60°C for 30 s. qRT-PCR was performed in triplicate for each sample and each sample had six biological replicates. The relative expression was estimated using the 2 ^-ΔΔCt^ method with the InM stage as calibration control (31), and statistical significance (*P* < 0.05) was determined using Student *t*-tests.

### 2.6 Absolute quantitation of *EcR* and *RXR* during the molting cycle

To quantify and compare the absolute expression level (number of gene copies) for *EcR* and *RXR* in various tissues among different molting stages, standard plasmid products of *EcR* and *RXR* were first prepared. The ORFs of *EcR* and *RXR* were cloned into the pMD-19-T vector (Takara, Dalian, China), and the vector was transformed into DH5α competent cell. Then, the plasmids were extracted as standards, and the copy number of *EcR* and *RXR* plasmids was calculated with the following formula: Plasmid copy number (copies/μL) = 6.02 × 10^23^ (copies/μL) × plasmid concentration (g/μL)/plasmid molecular weight (g/mol) (32). The standard curves for *EcR* and *RXR* plasmids were constructed using a 10-fold serially diluted standard plasmid with a known copy number calculated above. The correlation coefficient for the standard curve was greater than 0.99, and the amplification efficiency were between 0.95 and 1.05. The absolute quantitation PCR reactions of *EcR* and *RXR* at different molting stages were performed with a 25 μL reaction mixture, and the PCR procedure was conducted the same as the above relative quantitation experiment. cDNA of various tissues from different molting stages and standard plasmid products of *EcR* and *RXR* were quantified on the fluorescence quantitative PCR instrument at the same time and each sample had six biological replicates. The absolute quantitation expression level (copy numbers) of *EcR* and *RXR* from the collected tissues among different molting stages was measured based on the equation of linear regression of the standard curve.

### 2.7 Co-immunoprecipitation

To construct the required plasmids for the co-immunoprecipitation experiment, designed primers with Bam HI and Hind III restriction endonuclease sites were introduced according to the gene sequence of *EcR* and *RXR* (**Table S1**). FLAG-tag and HA-tag sequences were added to *EcR* and *RXR*, respectively. After PCR amplification, the EcR-FLAG and RXR-HA PCR products with Bam HI and Hind III endonuclease sites were obtained. Then, the PCR products of EcR-FLAG and RXR-HA were digested with double restriction enzymes (Bam HI and Hind III) and were cloned into pcDNA3.1(-) vector to obtain the pcDNA3.1-EcR-FLAG and pcDNA3.1-RXR-HA plasmids, respectively.

The constructed pcDNA3.1-EcR-FLAG and pcDNA3.1-RXR-HA plasmids were transfected into HEK293T cells. The transfected cells were harvested after 24 hours, washed with cold phos-phate-buffered saline (PBS) twice, and lysed with cell lysis buffer (Beyotime, Shanghai). The sampled cells were ultrasonic and centrifuged at the maximum speed for 10 mins. The supernatant was collected and incubated with FLAG beads at 4 °C overnight with gentle rotation. Then the FLAG beads were washed twice with lysis buffer, loading buffer was added and boiled for 10 mins. Finally, the western bolt experiment was conducted, and mouse anti-HA (Beyotime, AH158, 1:3000) and mouse anti-FLAG (Beyotime, AF519, 1:2000) monoclonal antibodies were used.

### 2.8 Dual-luciferase reporter assay

*E75* has been identified as a downstream responsive gene in the ecdysteroid signaling cascade, which is initiated by the EcR/USP heterodimer complex. The coding region and DNA sequence of the *E75* gene were obtained from the assembled genome of *E. sinensis* (26). Primer was designed to amplify the upstream 2,000 bp region of *E75*, which is usually considered as the promotor region. Then the *E75* promotor was digested by the double enzyme (NheI and HindIII) and inserted into the pGL3 vector. The constructed pGL3-E75 plasmid was used as a reporter vector in the dual luciferase reporter assay. The whole ORF of *EcR* and *RXR* with HindIII and BamHI restriction endonuclease sites were amplified and then digested with HindIII and BamHI enzymes. Later, the digested fragments were inserted into the pcDNA3.1(-) vector to construct the pcDNA3.1-EcR and pcDNA3.1-RXR expression plasmids.

The constructed pcDNA3.1-EcR, pcDNA3.1-RXR plasmids, the normalized vector (phRL-TK), and the reporter vector (pGL3-E75) were transfected into HEK293T cells seeded in 48-well plates according to the Lipofectamine 2000 manual. We designed five experimental groups: Group 1, pcDNA3.1 vector, pGL3 vector, and phRL-TK plasmid were transfected into HEK293T cells with the molar ratio of 24: 24: 2; Group 2, pcDNA3.1 vector, pGL3-E75, and phRL-TK plasmid were transfected into HEK293T cells with the molar ratio of 24:24: 2; Group3, pGL3-E75, pcDNA3.1-EcR, and phRL-TK were transfected into HEK293T cells with the molar ratio of 24: 24: 2; Group4, pGL3-E75, pcDNA3.1-RXR, and phRL-TK were transfected into HEK293T cells with the mole ratio of 24: 24: 2; Group5, pGL3-E75, pcDNA3.1-RXR, pcDNA3.1-EcR, and phRL-TK were transfected into HEK293T cells with the molar ratio of 24: 12: 12: 2. 24 hours after transfection, muristerone A (10 μm, Cayman, USA) was added into cell culture medium (33). Forty-eight hours after muristerone A added, the cells were washed twice with PBS. The enzymatic activities were measured by using Dual-Luciferase^®^ Reporter Assay System (Promega, USA) according to the instruction. The relative luciferase activity was determined by the normalized renilla luciferase activity (34). Each assay was performed in triplicate.

## 3 Results

### 3.1 The gene structure of *EcR* and *RXR* genes

**Figure 1:**
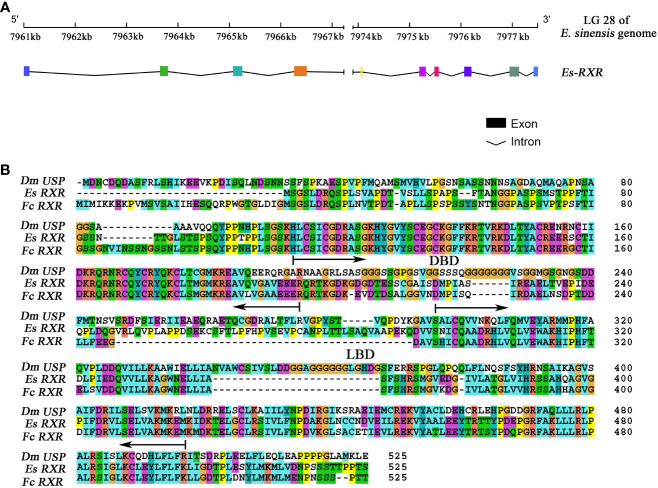
The gene structure characterization of *RXR* in *E. sinensis*. **(A)** The gene structure of *RXR*. Colored boxes indicated the different exons, and the black underline in the bottom figure denoted the intron boundary. **(B)** Amino acid sequence alignment of *RXR* with homologs from *F. chinensis* and *D. melanogaster*. Dm indicates *D. melanogaster*, Es indicates *E. sinensis* and Fc indicates *F. chinensis.* The following regions are underlined: DNA binding domain (DBD) (from 71 to 152) and ligand binding domain (LBD) (from 173 to 421).

*EcR* gene locates at Chromosome 26 of the *E. sinensis* genome, with 9 exons and 8 introns which were consistent with our previous study (accession number: KY303919) (35). *RXR* gene locates at the chromosome 28 of the *E. sinensis* genome, and contains 10 exons and 9 introns (**Figure 1A**, **Table S2**). The whole ORF sequence of *RXR* was verified by Sanger sequencing (accession number: MK604180).

The amino acid sequence alignment of RXR from *E. sinensis* with homologs from *F. chinensis* and *D. melanogaster* is presented in **Figure 1B**. Protein sequence alignment analysis showed that the sequence identity of RXR between *E. sinensis* and *C. sapidus*, *F. chinensis*, *D. magna*, *D. melanogaster*, *H. sapiens*, and *M. musculus* were 82.67%, 66.80%, 60.72%, 37.06%, 47.67%, and 47.39%, respectively. The DNA binding domain (DBD) and ligand binding domain (LBD) of RXR in *E. sinensis* were identified by searching the conserved domains in the NCBI database using CD-search algorithm (36). The lengths of DBD and LBD in *E. sinensis* were 82 (from 71 to 152) and 249 (from 173 to 421) amino acid residues, respectively. The sequence identity of RXR’s DBD between *E. sinensis* and *C. sapidus*, *F. chinensis*, *D. magna*, *D. melanogaster*, *H. sapiens*, and *M. musculus* was 92.68%, 95.12%, 89.02%, 89.02%, 79.27%, 81.71%, respectively. However, for the LBD, the sequence identity for the above comparison was 76.31%, 67.47%, 60.64%, 37.32%, 59.44%, and 58.23%, respectively.

### 3.2 Phylogenetic tree of *RXR* and protein structure prediction

**Figure 2:**
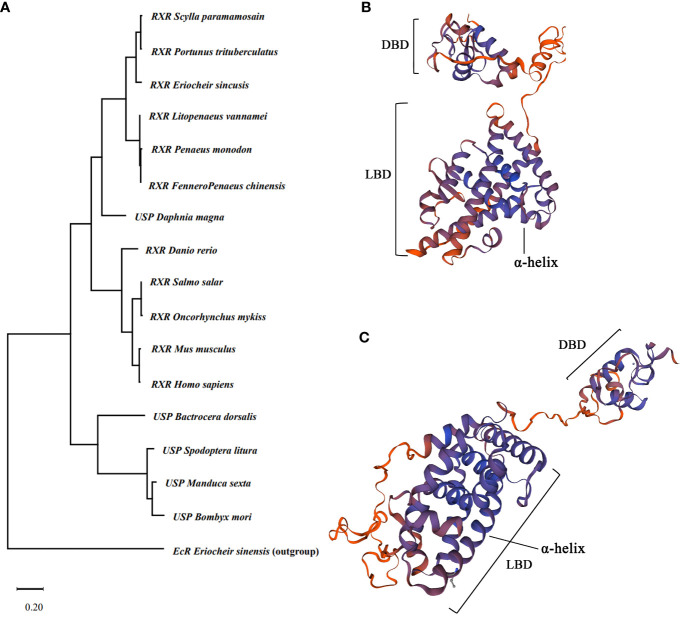
Evolutionary analysis of *RXR* gene and 3D protein structure prediction. **(A)** Maximum likelihood phylogenetic tree based on RXR amino acid sequences. The tree was generated by MEGA 11 with a bootstrap value of 1,000 bootstraps. The *EcR* of *Ericheir sinensis* was used as outgroup. **(B)** Predicted protein structure of EcR of *E sinensis.* **(C)** Predicted protein structure of RXR of *E sinensis*. DBD indicate DNA binding domain, and LBD indicate ligand binding domain.

The phylogenetic tree of RXR based on amino acid sequence could be separated into three clades, with RXR from crustacean and vertebrate species forming two clades and USP from insects forming another clade (**Figure 2A**). The phylogenetic tree may indicate the sequence divergence and functional difference of RXR and USP among crustaceans, insects, and vertebrates. Based on the predicted protein structure of EcR and RXR, typical features of nuclear receptors, such as the DNA binding domain and ligand binding domain, were identified (**Figure 2B, C**).

### 3.3 Spatial and temporal expression profiles of *EcR* and *RXR* during the molting process

**Figure 3:**
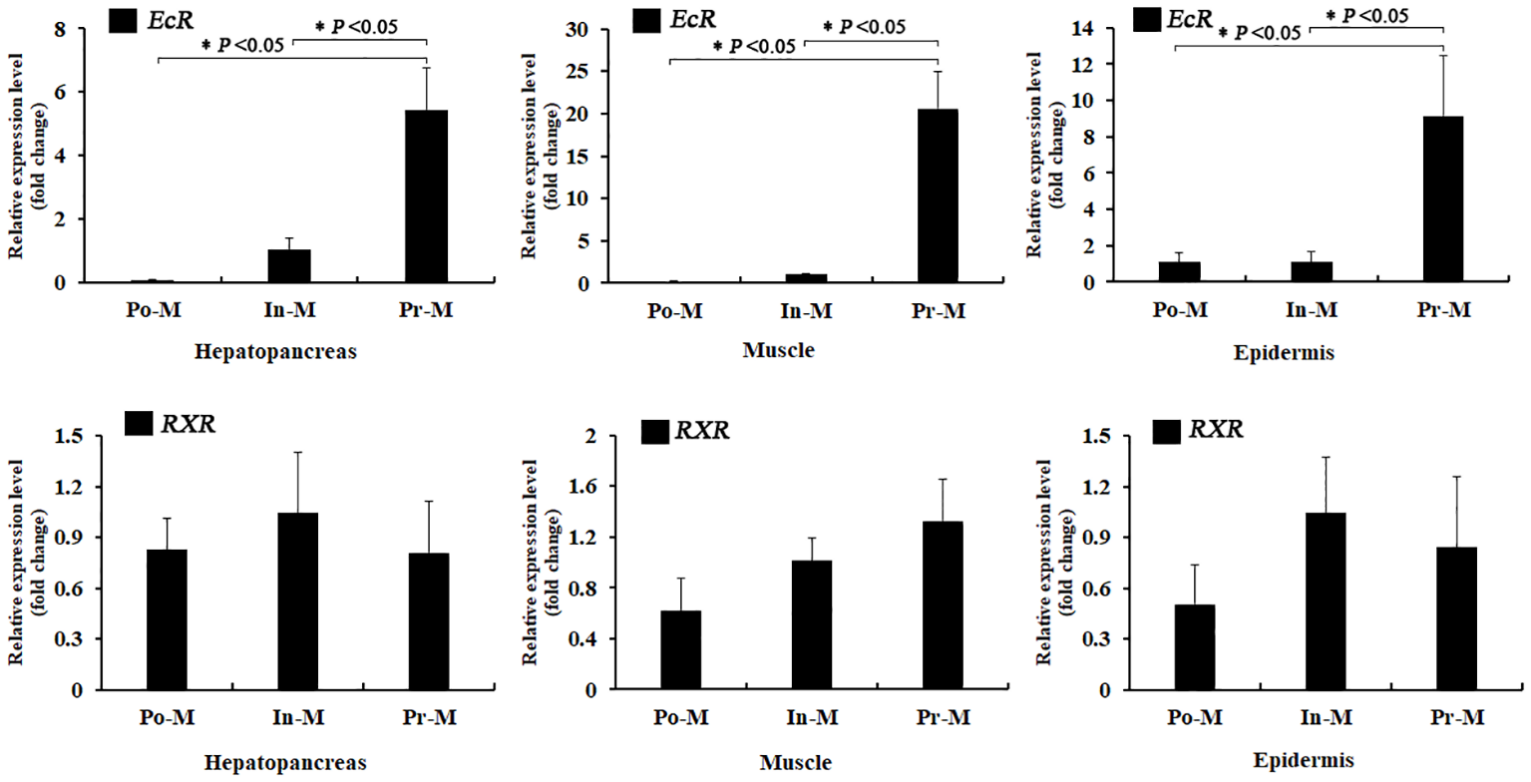
Relative expression analysis of *EcR* and *RXR* in different tissues and molt stages of *E. sinensis*. Po-M means postmolt, In-M means intermolt, and Pre-M mean premolt. Error bars represent standard error. “*” means significant difference (*p* < 0.05).

The qRT-PCR experiment indicated that both *EcR* and *RXR* were expressed in the hepatopancreas, epidermis, and muscle tissue during the whole molting process (**Figure 3**). Throughout the molting cycle, the expression of EcR exhibited dynamic fluctuations and increased significantly during the PrM stage in all three tissues. Specifically, the expression level of *EcR* was found to be 5.2 times, 8.3 times, and 20 times higher at the PrM stage compared to that at the InM stage in the hepatopancreas, epidermis, and muscle tissues, respectively (**Figure 3**). However, there was no significant expression difference for *RXR* in the three tissues during the molting process (*p* > 0.05) (**Figure 3**).

**Figure 4:**
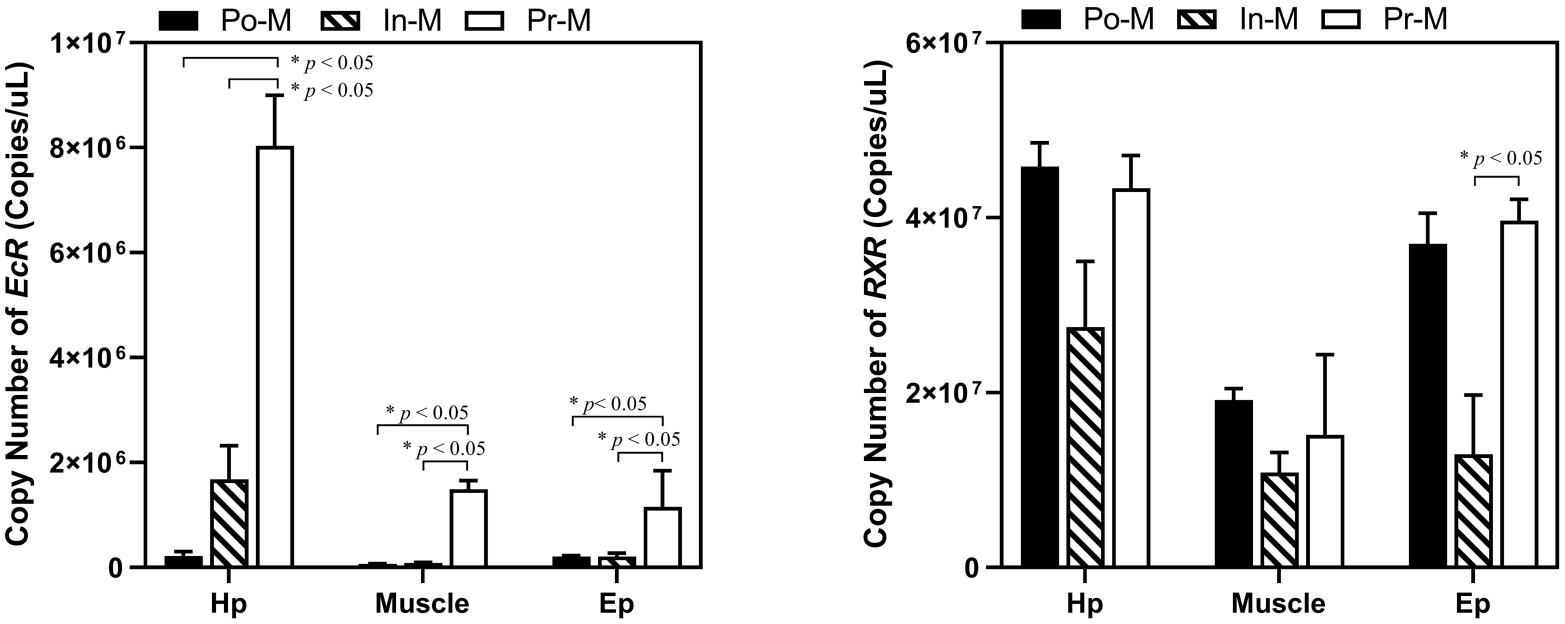
Absolute expression analysis of *EcR* and *RXR* in different tissues and molt stages of *E. sinensis*. Po-M means postmolt, In-M means intermolt, and Pre-M mean premolt. Error bars represent standard error. “*” means significant difference (*p* < 0.05).

The absolute expression level of *RXR* was significantly higher than that of *EcR* in all three tissues and molting stages (**Figure 4**). Specifically, the fold change of the expression level of *RXR*/*EcR* was 289 at PoM and 5 at PrM in hepatopancreas tissue, was 179 at PoM and 34 at PrM in epidermis tissue, and was 289 at PoM and 10 at PrM in muscle tissue. During the molting process, the absolute expression level of *EcR* showed dynamic expression and was significantly upregulated at the PrM stage in the three tissues. However, the absolute expression level of *RXR* was relatively stable, consistent with qRT-PCR results (**Figure 3**).

### 3.4 Molecular interplay of EcR and RXR proteins

**Figure 5:**
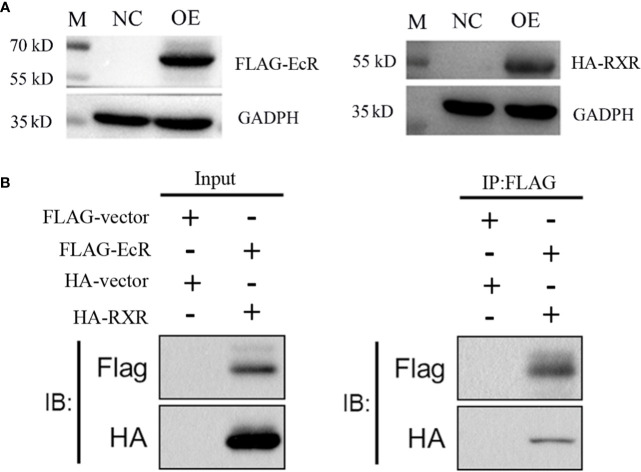
EcR interacts with RXR confirmed by a Co-IP assay. **(A)** Overexpression detection of EcR and RXR in 293T cells by Western blot. EcR-FLAG, RXR-HA, and control plasmids were overexpressed in 293T cells. M, marker. NC: negative control group. OE: overexpression group. The cells were harvested to verify the expression of EcR and RXR by Western blot. **(B)** The Co-IP experiment of EcR and RXR. The input and IP: Flag are labeled.

The overexpression experiment indicated that both EcR-FLAG and RXR-HA proteins were successfully expressed in 293T cells (**Figure 5A**). Regarding the Co-IP experiments, FLAG and HA proteins were successfully detected in the input group after overexpression, indicating successful overexpression of EcR-FLAG (~62 kDa) and RXR-HA (~52 kDa) in the 293T cells. In the IP group, flag protein was detected, indicating successful binding of flag beads with EcR-FLAG protein. Additionally, HA was also successfully detected, indicating that RXR-HA protein was pulled down by EcR-FLAG protein. (**Figure 5B**).

### 3.5 EcR interacts with RXR and regulates the transcription of *E75*

**Figure 6:**
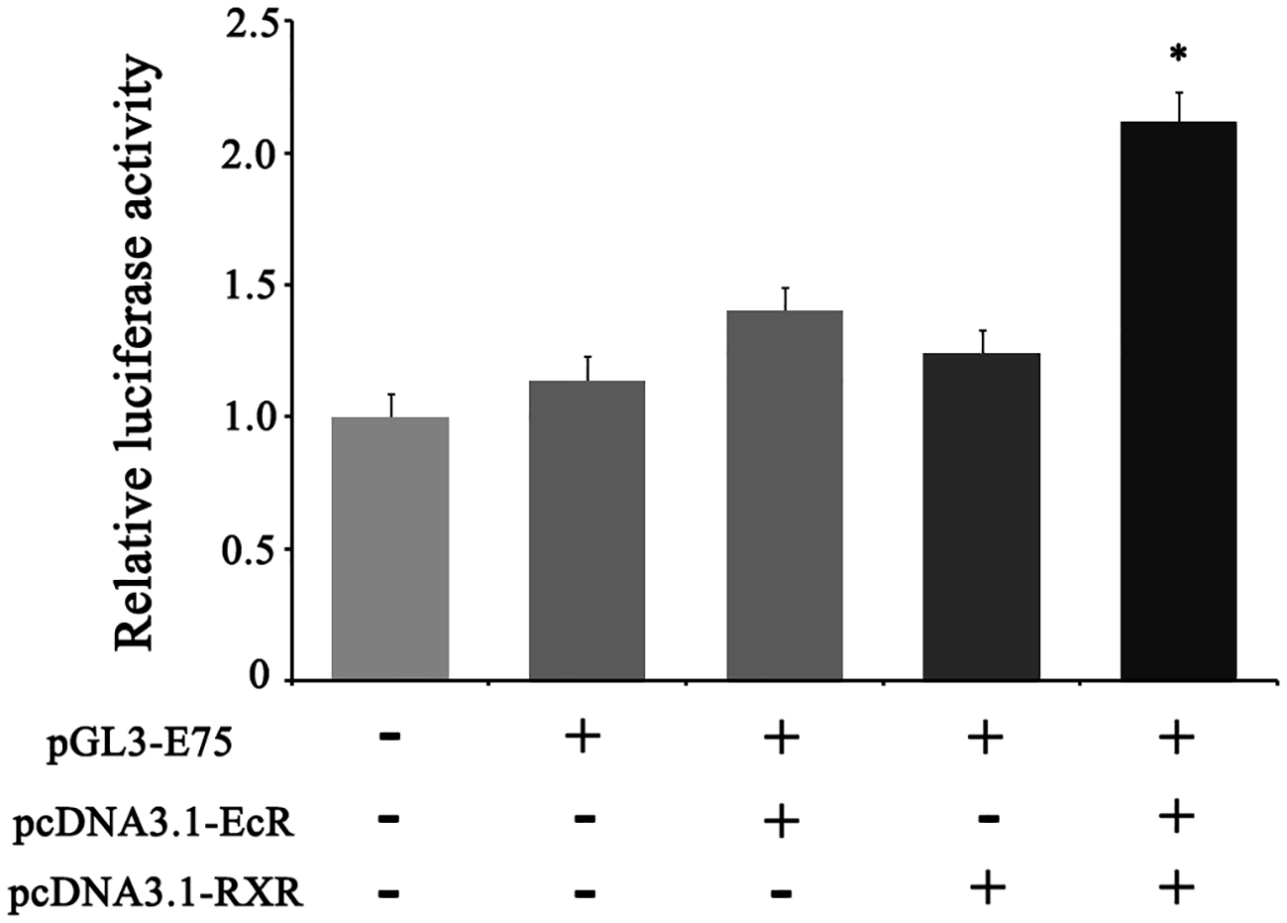
Transcriptional activity identification of *E75* in the HEK-293T cell line by dual-luciferase reporter assay. phRL-TK (internal control), pGL3-E75 (reporter plasmid), pcDNA3.1-EcR (overexpression plasmid of EcR), and pcDNA3.1-*RXR* (overexpression plasmid of RXR) were used in the transfection assay. The mole ratio of pGL3-E75, pcDNA3.1-EcR/pcDNA3.1-RXR, phRL-TK was 24: 24: 2 in the third and fourth column, and the mole ratio of pGL3-E75, pcDNA3.1-EcR, pcDNA3.1-RXR, phRL-TK was 24: 12: 12: 2 in the fifth column. Equal mole of empty pGL3 was used in the first column and its activity was defined as 1. Data represent mean ± SEM with three replicates. “*” means significant difference with other groups (*p* < 0.05).

The enzymatic activity of luciferase driven by the *E75* promoter was significantly higher than the control group (the empty pGL3 basic vector). Furthermore, in comparison to the transfection of pcDNA3.1-EcR (group 3) or pcDNA3.1-RXR (group 4) plasmid alone, co-transfection of pcDNA3.1-EcR and pcDNA3.1-RXR plasmids into HEK293T cells resulted in a significant increase in luciferase activity, indicating that EcR and RXR together induce the transcription of *E75* (**Figure 6**).

## 4 Discussion

Comparing the amino acid sequences of RXR in *E. sinensis* to those of other species, a higher sequence identity for the DBD of RXR than for the LBD was observed. This may illuminate why the target genes of RXR are almost identical, but the ligands of RXR are different among different organisms (37). For example, in insects, the USP, a homolog of RXR in crustaceans, does not respond to 9-cis-retinoic acid in cultured cells and tissues, while RXR can (38, 39). Moreover, phytanic acid and docosahexaenoic acid were identified as ligands of RXR, but not of USP (40, 41). These findings suggest that RXR and USP may have different LBD structures and ligands, indicating that the functions of EcR/RXR in crustaceans and EcR/USP in insects may differ to some extent.

In this study, the expression level of *EcR* increased sharply at the premolt stage, whereas the *RXR* presented a relatively stable expression level throughout the whole molting process (**Figure 3**). A consistent result was also identified in *D. melanogaster*, *P. japonicus*, and *F. chinensis* where the expression of *EcR* in hepatopancreas increased significantly from intermolt to premolt stage; however, the expression of *RXR* did not change much during the molting cycle (42–44). The research on the structure and function of the human *RXR* gene also indicated that *RXR* exhibits characteristics of a housekeeping gene (45). Moreover, the expression level of *RXR* was always much higher than that of *EcR* during the molting cycle (**Figure 3**). These results indicated that *RXR* may be a “constitutive-type” gene, similar to housekeeping genes, while EcR is a critical speed-limiting gene that plays an essential role in the molting process. Additionally, research on the structure of the promoter of the human RXRA gene has also indicated that RXR exhibits features of a housekeeping gene (45). It has been reported that RXR can dimerize with diverse nuclear receptors, such as FXR, PPARs and RARs, not only EcR, thus perform different functions other than molting regulation (46, 47). Regarding *E. sinensis*, RXR may also dimerize with other nuclear receptors to play different roles, which may explain the abundant and stable expression of *RXR* during the whole molting cycle. Taken together, our results suggest that *RXR* may be classified as a “constitutive-type” gene, similar to housekeeping genes, while *EcR* is a critical speed-limiting gene that plays an essential role in the molting process.

Research has indicated that EcR/USP complexes activate downstream gene expression in insects (48, 49). In *D. melanogaster*, several researchers have demonstrated that USP forms heterodimers with EcR to activate downstream gene expression, and USP is critical for EcR function (48, 50). As a dynamic complex, the activity of EcR can only be activated by binding with its ligands (5, 6, 8, 13, 51, 52). Studies in epithelial cells of *Chironomus tentans* have shown that EcR and USP form a unique complex that acts on palindromic structure and induce the transcription of *Hsp27* (10). In this study, EcR of *E. sinensis* can interact with RXR of *E. sinensis*, which was verified by the CO-IP experiment. Studies on *Neomysis integer* presented that EcR can also form heterodimer with RXR in GAL4 cell reporting system (53) and EcR and RXR of *Uca pugilator* formed heterodimer in S9 cell (54). These results strongly indicate that EcR may bind with RXR to form a functional heterodimer complex in crustaceans. Moreover, the transcriptional activity of downstream gene *E75* was significantly higher when RXR and EcR were both overexpressed than when only EcR or RXR was overexpressed in cells (**Figure 6**). Our result strongly indicates that the transcription of downstream genes, such as *E75*, in *E. sinensis* during the molting process can be activated by the functional heterodimer complex formed by EcR and RXR.

**Figure 7:**
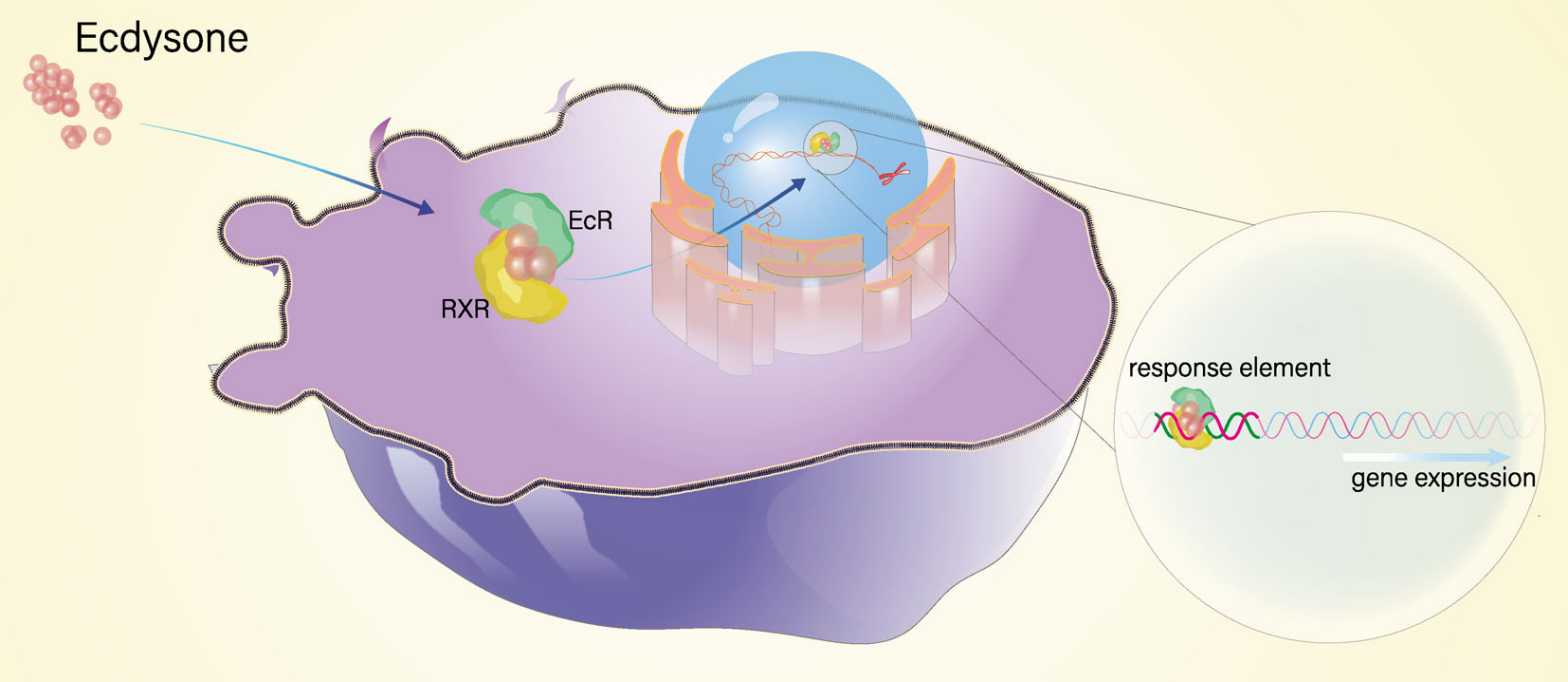
The schematic diagram of molecular interplay of EcR and RXR.

In conclusion, we clearly deciphered the gene structure of *EcR* and *RXR* and investigated the molecular interplay of them in the molting process in *E. sinensis*. Our results indicate that *EcR* is a key speed-limiting gene and *RXR* is a “constitutive-type” gene involved in the ecdysteroid signaling pathway. Moreover, EcR and RXR bind to form heterodimers to activate the transcription of downstream response gene *E75* to regulate the molting process of *E. sinensis* (**Figure 7**). Our research provides novel insights into the molecular mechanism of molting mediated by the ecdysteroid signaling pathway in crustaceans.

## Data availability statement

The original contributions presented in the study are included in the article/**Supplementary Material**. Further inquiries can be directed to the corresponding author.

## Ethics statement

The animal study was approved by Institutional Animal Care and Use Committee (IACUC) of Shanghai Ocean University. The study was conducted in accordance with the local legislation and institutional requirements.

## Author contributions

XC, JW and CW contributed to design the study. XC and XH contributed most analysis for the study. HY and HL helped to organized data. XC wrote the draft. XH, JW and CW contributed to draft review and editing. All authors contributed to manuscript revision, read, and approved the submitted version.

## Supplementary material

Supplementary Table 1 Primers used in this study for cloning and expression analysis of *Eriocheir sinensis EcR* and *RXR*.

Supplementary Table 2 The detailed position information of *EcR* and *RXR* in genome.
