# Supplementary material for: Molecular interplay between ecdysone receptor and retinoid X receptor in regulating the molting of the Chinese mitten crab, Eriocheir sinensis
Source: Front Endocrinol (Lausanne). 2023 Oct 19;14:1251723. doi: 10.3389/fendo.2023.1251723 (PMC10621794; doi:10.3389/fendo.2023.1251723)
Supplement: Supplementary Table 1 — Primers used in this study for cloning and expression analysis of Eriocheir sinensis EcR and RXR. [file Table_1.docx]

Table S1. Primers used in this study for cloning and expression analysis of *Eriocheir sinensis EcR* and *RXR*

| Primers | Primers sequences (5’-3’) | Targets |
| --- | --- | --- |
| RXR-ORF | F: ATGTCCGGCTCCCTGGATCG  R: CTAGCTGGTGGGGGGAGTGG | Amplify the whole ORF of *RXR* |
| EcR-ORF | F: TGTTTGTGTTGGGCTCTGGT  R: ATCCCAGATCTCAGCCAGGAA | Amplify the whole ORF of *EcR* |
| *RXR* | F: ACTGCTGCAATGACGTGGAA  R: GCTCGTCAGGGTAGGTGGTG | qRT*-*PCR for *RXR* |
| *EcR* | F: GACGGCACGTGTCGATGGAA  R: CGAGCTGGACACCTCGGAAC | qRT*-*PCR for *EcR* |
| *β*-actin | F: TCATCACCATCGGCAATGA  R: TTGTAAGTGGTCTCGTGGATG | Internal control |
| pcDNA3.1^-^-EcR | F: CGC**GGATCC**ATGGCCAAGGTGCTGGC  R: CCC**AAGCTT**TCAGTATCCAGAAACATCCCAGATCTCA | Construct plasmid |
| pcDNA3.1^-^-RXR | F: CGC**GGATCC**ATTATGGAAATGTCCGGCTCCCTGGATCG  R: CCC**AAGCTT**CTAGCTGGTGGGGGGAGTGG | Construct plasmid |
| *E75* | F: CTCAAGGAACCTCACAATGGC  R: TGCTCAGAATACACGGTTGCG | Amplify E75 |
| pGL3- E75 | F: CTA**GCTAGC**TCAAGGAACCTCACAATGGC  R: CCC**AAGCTT**TGCTCAGAATACACGGTTGCG | Construct plasmid |
| EcR-FLAG | F: CGC**GGATCC**GCCACCATGGCCAAGGTGCTGGC  R: CCC**AAGCTT**TCACTTGTCATCGTCATCCTTGTAGTCGATGTCATGATC  TTTATAATCACCGTCATGGTCTTTGTAGTCGTATCCAGAAACATCCCA | Construct plasmid |
| RXR-HA | F: CGC**GGATCC**GCCACCATGTCCGGCTCCCTGGATC  R: CCC**AAGCTT**CTAAGCGTAGTCTGGGACGTCGTATGGGTAGCTGGTGG  GGGGAGTGGTGCT | Construct plasmid |

Note: The underlined nucleotides are nucleotides and the boldfaced nucleotides is enzyme digestion site sequence.
