# Supplementary material for: Molecular interplay between ecdysone receptor and retinoid X receptor in regulating the molting of the Chinese mitten crab, Eriocheir sinensis
Source: Front Endocrinol (Lausanne). 2023 Oct 19;14:1251723. doi: 10.3389/fendo.2023.1251723 (PMC10621794; doi:10.3389/fendo.2023.1251723)
Supplement: Supplementary Table 2 — The detailed position information of EcR and RXR in genome. [file Table_2.docx]

Table S2. The detailed position information of *EcR* and *RXR* in genome

| Updated *EcR* structure | LG26 | | *RXR*  structure | LG28 | |
| --- | --- | --- | --- | --- | --- |
|  | Start | End |  | Start | End |
| Exon 1 | 6556773 | 6557226 | Exon 1 | 7961023 | 7961132 |
| Exon 2 | 6981746 | 6981919 | Exon 2 | 7964081 | 7964236 |
| Exon 3 | 6983732 | 6983872 | Exon 3 | 7965758 | 7965938 |
| Exon 4 | 6984314 | 6984450 | Exon 4 | 7966956 | 7967198 |
| Exon 5 | 6985202 | 6985282 | Exon 5 | 7974492 | 7974527 |
| Exon 6 | 6986598 | 6986723 | Exon 6 | 7975577 | 7975703 |
| Exon 7 | 6991915 | 6991990 | Exon 7 | 7975871 | 7975953 |
| Exon 8 | 6994276 | 6994498 | Exon 8 | 7976450 | 7976591 |
| Exon 9 | 6996310 | 6996557 | Exon 9 | 7977332 | 7977516 |
|  |  |  | Exon 10 | 7977794 | 7977880 |
